# Executive Function in Autism Spectrum Disorder: History, Theoretical Models, Empirical Findings, and Potential as an Endophenotype

**DOI:** 10.3389/fpsyt.2019.00753

**Published:** 2019-11-11

**Authors:** Eleni A. Demetriou, Marilena M. DeMayo, Adam J. Guastella

**Affiliations:** Autism Clinic for Translational Research, Brain and Mind Centre, Faculty of Medicine and Health, Children’s Hospital Westmead Clinical School, University of Sydney, Sydney, NSW, Australia

**Keywords:** executive function, autism spectrum disorder, neurobiology, excitation/inhibition, GABA, endophenotype

## Abstract

This review presents an outline of executive function (EF) and its application to autism spectrum disorder (ASD). The development of the EF construct, theoretical models of EF, and limitations in the study of EF are outlined. The potential of EF as a cognitive endophenotype for ASD is reviewed, and the Research Domain Criteria (RDoC) framework is discussed for researching EF in ASD given the multifaceted factors that influence EF performance. A number of executive-focused cognitive models have been proposed to explain the symptom clusters observed in ASD. Empirical studies suggest a broad impairment in EF, although there is significant inter-individual variability in EF performance. The observed heterogeneity of EF performance is considered a limiting factor in establishing EF as a cognitive endophenotype in ASD. We propose, however, that this variability in EF performance presents an opportunity for subtyping within the spectrum that can contribute to targeted diagnostic and intervention strategies. Enhanced understanding of the neurobiological basis that underpins EF performance, such as the excitation/inhibition hypothesis, will likely be important. Application of the RDoC framework could provide clarity on the nature of EF impairment in ASD with potential for greater understanding of, and improved interventions for, this disorder.

## Preface

Autism spectrum disorder (ASD) is a neurodevelopmental condition defined by difficulties in social communication and interaction, as well as restricted, repetitive patterns of behavior, interests, or activities (1). The social communication domain includes difficulties in reciprocal social interaction (2); deficits in non-verbal social communication (3, 4); and impairments in ability to develop, maintain, and understand relationships (5). Symptoms associated with the restricted and repetitive behavior domain manifest across motor, verbal, non-verbal, and sensory modalities (6). Observed behaviors in the restricted and repetitive domain may include motor stereotypies, echolalia, insistence on sameness, ritualized behaviors, narrow interests, and hyper- or hypo-reactivity to sensory stimuli (1).

A number of cognitive models (5, 7) have been proposed to explain difficulties observed across the life span in ASD (8, 9). One such model, the executive dysfunction hypothesis, focused on explaining the atypical executive function (EF) processes in ASD (10, 11). This model developed following observation of difficulties in set shifting (ability to shift mindset to new concepts), response inhibition (ability to inhibit a dominant response), and working memory (retaining and updating information in short-term memory) (12). Early research focused on set shifting (13) and its relationship to stereotypic and repetitive behaviors (14). Findings were interpreted to show a link between cognitive rigidity and the perseverance to routines and stereotypies observed in ASD (15). Increasingly, however, research implicates a broader influence of EF on the ASD phenotype. These include impacts of EF on social cognition (16, 17), mental health (18), disability (19, 20), and lifelong functioning outcomes (21). Overall, findings on EF in ASD suggest a broad impairment (22, 23) that is characterized by marked heterogeneity (24). The study of EF in ASD has focused primarily on investigating discrete EF constructs or domains (25). This is in contrast to the wider range of EF models developed in response to neurotypical development (26–28).

This paper presents a discussion of EF research in ASD, an overview of EF models drawn from typical and atypical development, and their potential contribution to the study of ASD. Factors that may moderate research outcomes of EF in ASD are also discussed. These include measurement issues of the EF construct, moderator influences on EF, and differences in the developmental trajectory of EF in ASD. Finally, a research model based on the efficacy of EF as an endophenotype is proposed within the research framework of Research Domain Criteria (RDoC) (29).

## Conceptualization of Executive Function

The term EF was first proposed in the mid-20^th^ century to explain functions associated with the frontal cortex (30). Frontal lobes were of interest following case studies, such as Phineas Gage (31), where it was observed that frontal lobe damage was associated with impairment of discrete functions, such as planning, organization, and self-regulation, even though general intellectual functioning remained mostly intact. This observation and subsequent case studies (32) led to conclusions that the frontal lobes have a primary role in organizing higher-order functions (33). Much of the subsequent research of EF focused primarily on the frontal lobes and functions associated with them (34, 35).

EF has been broadly defined as the overarching regulation of goal-directed, future-oriented, higher-order cognitive processes (28, 36–38). Although there is general agreement on the broad concept of EF, the theoretical models and processes that may underpin it vary considerably. Models of EF draw on different theoretical paradigms and include cognitive, clinical, behavioral, and neurobiological frameworks (39). This has, in part, contributed to the divergent frameworks of theorized models and mechanisms (38). In this paper, we present an overview of EF models and distinguish between them based on the level of analysis and measurement of the EF construct. Models are classified based on behavioral, cognitive, neuroanatomical, and neural measurement frameworks. **Tables 1** and **2** summarize key features of these EF models and associated measurement tools.

**Table 1:** Summary of EF models.

EF model	EF construct(s)	EF mechanism	Neurobiological underpinnings	Predictions	Interventions
***Unifactor models***					
Working memory (40)	Central executive Central executive fractionated to component parts of – Focused attention – Divided attention – Attention switching – Interface with long-term memory (episodic buffer)	Attentional focus, storage, and decision making Central executive regulates information control to the working memory component process of the phonological loop and visuospatial sketchpad Information integrated in episodic buffer and interfaced with long-term memory	Baddeley (40) noted that this has been guided by observations of patients with neurobiological damage but does not specify distinct neurobiological mechanisms	Model viewed as a homunculus approach predicting complex behavior regulation Impaired mechanisms would lead to broad behavioral dysregulation	Working memory assessment system for children with a practical guide for cognitive interventions (40, 41)
Attentional control (42)	Executive attention	Fractionation of attentional system into components of – Orienting – Alerting – Cognitive Cognitive attention responsible for regulation of cognitive functions		Impaired mechanisms would lead to broad behavioral dysregulation	Cognitive remediation programs to improve attentional control
Supervisory Attentional system (43)	Executive attention Inhibitory control	Distinction is made between routine or habituated actions versus non-routine actions Non-routine actions require the individual to disengage from habituated behavior patterns and make a novel response The supervisory attentional system exerts supervisory control in novel situations where routine or previously learned behaviors must be inhibited		Impaired mechanisms would lead to broad behavioral dysregulation including perseverative behaviors, distractibility, and apathy due to disrupted inhibitory control (44)	Cognitive remediation programs to improve attentional control
***Multifactorial models***					
Unity and diversity (12)	Common factor (response inhibition) Set shifting Updating/working memory	Maintain and manage goals Task switching Updating and replacing irrelevant information in working memory	Genetic underpinning of EF common factor (45) Frontal lobe involvement for common EF factor; prefrontal cortex and basal ganglia circuitry for shifting factor; basal ganglia mediated updating process (46) Mediated by GABA/glutamate neural mechanisms (47)		Pharmacological interventions targeting GABA and cognitive interventions addressing the specific cognitive mechanisms
***Fractionated models of EF***					
	Set shifting_1, 2_ Response inhibition_1,2_ Working memory_1,2_ Planning_1_ Problem solving_1,2_ Reasoning_1,2_ Fluency_2_ Categorical processing_2_ Verbal abstraction_2_	Regulation of discrete EF cognitive processes	Neurobiological underpinnings not specifically defined in the model but supported by findings of neuroanatomical localization of discrete domains and functional connectivity between brain regions The Delis–Kaplan model draws on observations of patients with prefrontal lobe injuries, and emphasis is therefore on the prefrontal lobes	Impairment in discrete EF processes	Cognitive remediation interventions addressing each EF domain Pharmacological interventions addressing neural substrates
Diamond’s model of EF_1_ Delis–Kaplan model of EF_2_						
***Models linking EF and behavioral regulation***					
Stuss’ model of EF (28)	Task setting Task monitoring	EFs interacting with non-EF domains of: Energization Behavioral/emotional self-regulation Metacognition	Task setting: left lateral frontal cortex Task monitoring: right lateral frontal cortex Behavioral/emotional regulation: orbitofrontal cortex Energization: superior medial prefrontal cortex Metacognition: frontal poles	Impairment in EF processes of task setting and monitoring leading to specific deficits and overall dysregulation due to association with behavioral/emotional self-regulation	Cognitive remediation interventions targeting EF processes and potentially pharmacological interventions targeting underpinning neural mechanisms
Barkley’s model of EF	**“the use of self**-**directed actions so as to choose goals and to select, enact and sustain actions across time towards those goals usually in the context of others often relying on social and cultural means for the maximization of one’s long**-**term welfare as the person defines that to be” (** 37 **)**	Mediated by cognitive processes that tap into traditional definitions of EF Self-directed attention (self-awareness and monitoring) Self-restraint (inhibition) Self-directed sensing (non-verbal working memory) Self-directed speech (verbal working memory) Self-directed emotions and motivations Self-directed play (planning and problem solving)	Development of five EFs draws on Luria’s model and observations of patients with prefrontal lobe injuries (37)	Impaired regulation of each of the domains leading to overall difficulties in goal attainment	Intervention strategies may be addressing distinct underlying cognitive components of each of the self-management domains
Gioia’s model of EF	Self-regulation of behavior based on “selection, initiation, execution and monitoring of cognition and behaviour,” p.1 (48)		“Frontal systems” regulation of EF processes Emphasis on the regulatory control by the frontal lobes of cortical and subcortical areas, p.3 (48)	Impaired regulation of each of the domains	Intervention strategies may be addressing distinct underlying cognitive components of each of the self-management domains
***Neurobiological models of EF***					
Luria’s model	Complex information processing	Functional integration of three brain functional units First and second functional units: responsible for alertness and sensory information processing Third functional unit: responsible for regulation and execution of behavior	First and second functional units controlled by parietal, temporal, and occipital lobes Third functional unit regulated by the frontal lobes	“Frontal lobe syndrome” (49) Disinhibition Inability to follow sequence of action Repetitive motor movements	
E/I hypothesis		GABA/glutamate balance	Neural circuitry cortical and subcortical areas	Impairment in discrete EFs depending on neuroanatomical localization	Pharmacological interventions

EF, executive function; E/I, excitation/inhibition; GABA, *γ*-aminobutyric acid.

**Table 2:** The definition and assessment measures of discrete EF domains.

EF domain	Neuropsychological and experimental task measures
Set shifting/concept formation Set shifting or concept formation is defined as the capacity to shift between mental processes to form new concepts and identify the conceptual relationships shared by stimuli (12, 50). Other commonly used terminology for set shifting includes concept formation and cognitive or mental flexibility (25). Theorized mechanisms for set shifting have included switching between mental processes. It has been argued, however, that set switching (51) represents a distinctly different EF component that needs to be differentiated from set shifting.	Wisconsin Card Sorting Test (WCST) (52) Intra/Extra Dimensional Shift (IED)—CANTAB (53) Sorting test—D-KEFS (50) Dimensional Change Card Sort test (DCCS) (54) DCCS—NIH Cognition ToolBox (55) Flexible Item Selection Task (FIST) (56). Set Shifting test—CogState (https://www.cogstate.com/) Rule Shift Cards test—BADS (57) Temporal Judgement test—BADS (57)
Mental flexibility/set switching Set switching has been defined as the capacity to switch between mental processes (multiple tasks, operations, or mental sets) in response to changing demands (51, 58). It is distinct from set shifting, where the focus is on identifying novel relationships.	Trails Making Test (Trails B) (59). Trails Making Test—D-KEFS (50)
Fluency Fluency is defined as the capacity to generate verbal and non-verbal stimuli including ideas, designs (50), and words (60). Verbal fluency is a frequently studied measure of executive functioning (34) and is distinguished into phonemic (generativity for unrelated words) and semantic fluency (generativity for semantically related words or categories) (61). There is some debate as to whether phonemic and semantic fluency represent EF (36, 60) or language processes (62). However, a number of studies supported by neuroimaging findings (34) suggest that verbal fluency is reliant on core EF processes (63, 64).	Controlled Oral Word Association Test (COWAT) (65) Verbal Fluency test—D-KEFS (50) Design Fluency test—D-KEFS (50). 20 Questions Test—D-KEFS (50) Word Context test—D-KEFS (50) Proverb test—D-KEFS (50)
Planning Planning is defined as the capacity to execute a sequence of actions so that a desired goal is achieved (36).	Tower of Hanoi (66) Tower of London (67) One Touch Stockings (OTS)—CANTAB (53) Stockings of Cambridge (SOC)—CANTAB (53) Action Programme Planning test—BADS (57) Key Search test—BADS (57) Zoo Map test—BADS (57) Modified Six Elements test—BADS (57)
Response inhibition Response inhibition primarily refers to the ability to inhibit a previously learned or prepotent response (12). Two additional components contribute to inhibition: resistance to distractor interference and resistance to proactive interference (68). Resistance to distractor interference refers to the ability to process a target stimulus while ignoring irrelevant information presented at the same time, while resistance to proactive interference refers to the ability to efficiently process distractors from recently activated memory stimuli. Some research classifies resistance to proactive interference as a working memory process.	Stroop test (69) Color-Word Interference Test—D-KEFS (50) Go/no-go task (70) Hayling test (71) Eriksen flanker task (72). Stop Signal Task—CANTAB (53) Flanker Inhibitory Control and Attention test—NIH Cognition ToolBox (55) Go–No Go Test—CogState (https://www.cogstate.com/)
Working memory The concept of working memory is sometimes used interchangeably with short-term memory (STM), although different processes relate to each. Working memory refers to the capacity to store and dynamically manipulate information in temporary STM (36).	Letter sequencing task (73) Digits Backwards—Wechsler Memory Scale (74) Spatial Working Memory (SWM)—CANTAB (53). Spatial Span (SSP)—CANTAB (53) List Sorting Working Memory Test—NIH Cognition ToolBox (55) n-back task (75) One Back test—CogState (https://www.cogstate.com/) Two Back test—CogState (https://www.cogstate.com/)
Hot EF Top–down processes activated in situations with motivational and emotional significance (76).	Affective Go/No-go (AGN)—CANTAB (53) Cambridge Gambling Task (CGT)—CANTAB (53) Information Sampling Task (IST)—CANTAB (53) Iowa Gambling test (77)
Emotional/personality change, motivational change, behavioral change, cognitive change	Dysexecutive Questionnaire (DEX)—BADS (57)
Global executive composite Behavioral Regulation Index—initiate, organization of materials, plan/organize, task monitor, working memory Metacognition Index—emotional control, inhibit, self-monitor, shift	Behavioral Rating Inventory of Executive Function (BRIEF) (48)
Self-management in time, self-organization/problem solving, self-restraint, self-motivation, self-regulation of emotion	Barkley Deficits in Executive Functioning Scale (BDEFS) (37)

BADS, Behavioural Assessment of the Dysexecutive Syndrome; CANTAB, Cambridge Neuropsychological Test Automated Battery; D-KEFS, Delis–Kaplan Executive Function System.

### Cognitive and Behavioral Models of EF

A number of cognitive models of EF have discriminated between automatic and controlled cognitive processes (78) that are regulated by discrete attentional systems. Models focusing on attentional control included those proposed by Baddeley (79), Posner (42), and Shallice (80). Executive attention was attributed a regulatory role that facilitated focus on salient cues and regulated EF processes (**Table 1**).

Many researchers adopted a fractionated approach in order to distinguish between individual EF processes or domains (EFs) (12, 81) (**Table 2**). The number of discrete EFs reported on in the literature has ranged from 2 (82) to more than 30 (38). The three most commonly reported or core EFs are set shifting, response inhibition, and working memory (12, 38). Different levels of complexity have been proposed for EFs. For example, it is suggested that the three core EFs above, contribute to the higher-order EFs such as reasoning, planning, and problem solving (81). The Delis–Kaplan model (50) was developed in response to clinical observations of functions sensitive to frontal lobe damage and proposed nine EFs (**Table 1**). Until recently, most research focused on the study of a combination of the above core and higher order EFs. These are broadly referred to as cool EFs, defined as EF processes that are conducted independently of contextual framework or affective and motivational influences (83).

More recently, a distinction has been drawn between cool EFs and other cognitive processes, defined as hot EFs (84). Hot EFs are defined as the cognitive processes mediated by affective and motivational demands (76). They represent goal-oriented behaviors, moderated by personal appraisal of the affective or motivational significance of the stimuli. Hot EFs are increasingly studied in ASD cohorts (85) and are particularly relevant for this group because of their likely influence on behavioral regulation (86). Behavioral regulation is an integral component of models proposed by Stuss (28, 87), Barkley (37), and Gioia (48). Each of these models adopts a multifactorial approach that integrates cool and hot EFs as well as behavior regulatory control to varying degrees.

The model proposed by Stuss (28, 87) integrates cool EFs (task setting and monitoring) and non-EFs frontal lobe processes (energization, behavioral/emotional self-regulation, and metacognition). Energization refers to processing speed when completing cognitive tasks. Behavioral/emotional self-regulation is in part dependent on activation of EFs (task setting and monitoring). Metacognition has a higher-order supervisory role in integrating all EFs and non-EFs processes towards goal attainment.

Barkley’s model (88) is defined by five EF factors that regulate behavior towards achieving future goals (37). The five EF factors were empirically derived from behavioral ratings, primarily in cohorts with attention deficit hyperactivity disorder (ADHD). They are described as an individual’s ability to manage time, organize and problem-solve, exercise restraint, self-motivate, and regulate emotion (37). The five EF factors are surmised to be influenced by external (cultural/societal factors) and intra-individual processes (88).

Gioia and associates (48) utilized the umbrella definition of self-regulatory process of EF that involves the “selection, initiation, execution and monitoring of cognition and behaviour” (p. 1). Within this framework, they developed a behavioral assessment that utilizes self- and/or informant ratings and draws on cool EFs (e.g. response inhibition, set shifting, and working memory) and behavioral control (e.g. emotional control).

### Neurobiological and Neural Models of EF

Alexander Luria was one of the first researchers to introduce a model based on neurobiological processes (26) suggesting the broader engagement of various brain regions. In this model, frontal lobes were conceptualized as the regulatory area directing complex problem solving. Damage to the frontal lobes was associated with the frontal lobe syndrome (49), characterized by disinhibition, inability to follow a sequence of instructions, and repetitive motor movements.

Advancements in neuroimaging techniques have placed increasing focus on neuroanatomical localization of EF processes primarily within frontal cortical regions. Localization of cool EF processes has been associated primarily with the dorsolateral prefrontal cortex (PFC), while the top–down processes that regulate hot EFs are linked to the orbitofrontal or ventromedial prefrontal cortex. Some cognitive models also propose specific neuroanatomical correlates of EF. For example, for Stuss' model (87) it was proposed that the task setting and monitoring EFs are localized in the left and right lateral frontal cortex, respectively, while behavioral/emotional regulation corresponds with the localization of hot EFs in the orbitofrontal cortex. Energization is reported to be mediated by the superior medial prefrontal cortex, while metacognition is guided by the frontal poles (87).

The identification of these regional contributions, while valuable, does not encapsulate the broad cortical systems that are being recognized as significant in the neural processes that underlie EF processes (89). Building on the neuroanatomical localization of EF, connectivity models focus on neural circuitry between cortical regions and may present a more integrated approach in the study of EF.

Neuroimaging studies identified that discrete EFs are linked to broader brain networks including the areas within the prefrontal cortex. For example, set shifting was associated with activity of the lateral prefrontal cortex, anterior cingulate cortex, and inferior parietal lobule (52). Set switching task was associated with involvement of the prefrontal cortex and frontoparietal areas of the brain (52, 90). An extended brain network connectivity between dorsal and ventral brain networks was observed in fluency tasks including activation in the inferior frontal gyrus and left dorsolateral prefrontal cortex (34). A differentiation between dorsal and ventral brain networks was observed between phonemic and semantic fluency tasks, respectively (61). Similarly, extended brain network involvement is reported during completion of planning tasks including activation of the dorsolateral prefrontal cortex, the anterior and posterior cingulate areas, and the parietal cortex (91). Activation of frontal regions during working memory tasks included activation of the bilateral superior and middle frontal gyri, bilateral frontal polar regions, and precuneus gyrus (92).

At the neurochemical level of analysis, a number of neurotransmitters have been linked to EF processes. A comprehensive review (93) summarized the role of four neurotransmitter systems in EF. Dopamine (DA) was reported to influence cool EF constructs (set shifting, response inhibition) and to moderate hot EF reward processes. Norepinephrine (NA) circuits were associated with a number of EF cognitive processes (including response inhibition and set shifting likely due to influence of NA on arousal and attentional systems. Serotonin (5-hydroxytrypatamine [5-HT]) modulated response inhibition, through its action in the orbitofrontal cortex. Finally, the cholinergic system mediated set shifting and was proposed to also interact with a number of other neural circuits for a more complex integration of EF processes.

The role of γ-aminobutyric acid (GABA) is increasingly linked with mediating processes associated with neural circuitry in the prefrontal cortex. GABA is the primary inhibitory neurotransmitter in the mature brain, working with excitatory glutamate to create an excitation/inhibition (E/I) balance thought to reflect the activity of the cortex. More excitation is theorized to represent greater activity, while greater inhibition suggests decreased cortical activity (94). Increased GABA (compared to glutamate) within the lateral PFC has been associated with better ability to select between competing tasks (95). Improved working memory performance under increased memory load was associated with higher GABA concentration in the dorsolateral PFC. A recent study (pre-print) (47) attributed a key role to GABAergic genetic contributions to the common EF factor (45), using a large sample in a genome-wide association study (GWAS). This study highlighted the role of the excitatory/inhibitory balance in EF, especially the role of GABA-mediated inhibition.

The models described above reflect the divergent approaches taken in the study of EF in normative literature. In ASD, however, focus has been primarily on comparing diagnostic groups with autism and other cohorts on performance on discrete EF constructs. The executive dysfunction hypothesis discussed below sums a large part of empirical research of EF in ASD. It may reflect efforts to identify discriminating profiles between different groups. Novel approaches to the study of EF in ASD have focused on brain connectivity and neurotransmitter imbalance with limited evaluation of other EF models.

## Executive Function and Autism Spectrum Disorder

### Executive Dysfunction Hypothesis

Early studies of EF in ASD were summarized in a review by Pennington and Ozonoff (10). Executive dysfunction was proposed as a model for understanding behavioral problems in ASD, including impaired theory of mind (ToM). Their review of research studies across neurodevelopmental disorders suggested that discrete EFs (set shifting, response inhibition, and working memory) might be appropriate cognitive markers for differentiating between ASD and ADHD.

Empirical findings on EF deficits in ASD were subsequently formalized in the executive dysfunction hypothesis (25) proposed in an effort to review and integrate the extant literature of EF in ASD. The review focused on four EFs: planning, mental flexibility, inhibition, and self-monitoring, assumed to represent the core EF domains. The executive dysfunction hypothesis suggested impairment on distinct EF domains, supporting a fractionated model of EF. In addition to identifying impairment in EFs, the review also highlighted considerable variability in EF performance between studies and within cohorts.

Since the introduction of the executive dysfunction hypothesis, there has been a proliferation of studies investigating cool EFs in ASD; these have been synthesized in a number of meta-analyses. Findings in the extant literature of executive dysfunction and heterogeneity in EF performance complement the observations made by Hill (25) and Pennington (10).

A meta-analysis on cognitive flexibility (96) indicated life span impairment in ASD. The study adopted a broad definition of cognitive flexibility and combined research on set shifting, set switching, and inhibitory control. A meta-analysis in children and youth investigating the components of response inhibition, prepotent response inhibition and interference control, identified age related differences (68). Impairment in prepotent response inhibition attenuated with increasing age, whereas difficulties in interference control persisted across the life span. An investigation of working memory (97) in children and young adolescents revealed impairment across both verbal and spatial working memory. There were no age-related differences; however, a larger effect size was observed for spatial compared to verbal working memory, suggesting greater difficulties in the spatial domain for youth with ASD. Planning is considered a key EF in adaptive behavior, and a meta-analysis reported impairment in planning for individuals with ASD (98). Planning difficulties were independent of moderator influences of age, intellectual functioning, and assessment type. The meta-analyses described above confirm impairment in discrete EFs; however, it remains uncertain whether these are underpinned by a common mechanism or whether discrete EFs are differentially impaired in ASD.

Two recent meta-analyses (22, 23) investigated cool EFs in ASD across multiple EF domains and thus address this question. Broad impairment in EF was observed both in children and youth (22) and across the life span (23). In the (22) meta-analysis, impairment in response inhibition and planning was less prominent compared to deficits in flexibility (set switching and set shifting), generativity/fluency, and working memory. Impairment across all of the above domains was identified in the (23) meta-analysis. Both studies suggest that an underlying common pathway may influence EF processes in ASD.

The meta-analyses described above also identified substantial heterogeneity in EF performance, despite consideration of a number of moderator variables. Hot EFs may be contributing to the unexplained heterogeneity, particularly in the view that they are independent of cool EF processes (86, 99). Comparable to most research of cool EFs in ASD, the study of hot EFs principally adopted the fractionated approach investigating discrete domains. Impairment has been observed in tasks associated with affective decision making and delay discounting (85, 100). Given the limited studies completed to date, it is unclear whether hot EFs could alone explain the heterogeneity observed in EF performance in ASD.

### Atypical Brain Connectivity

Throughout the ASD literature, there have been consistent findings of atypical functional connectivity, though this has varied between over-connectivity and under-connectivity (101). The regions impacted in ASD include areas encompassed by the default, salience, and executive control networks (102) and in cortical–subcortical circuitry (103). When connectivity is investigated for distinct EFs, there are reported differences in the circuitry associated with working memory (104) and response inhibition (105), with atypicalities reported to persist across the life span (106).

### The Excitation/Inhibition Hypothesis (E/I)

The E/I model (107) examines observed behavior in ASD at the neural level. The E/I model focuses on the action of glutamate and GABA and the balance between the two. The model suggests that an imbalance between neural excitation (driven by glutamate) and neural inhibition (driven by GABA) in brain circuits contributes to ASD symptomatology (108) and associated impairment in perceptual, motor, and cognitive systems (107, 109). The links between ASD, EF, and the E/I hypothesis have not been extensively investigated. The observed reductions in GABA concentration and GABA receptors in the frontal lobes (110) suggest a likely influence of GABA on frontal lobe processes, including EF. For example, greater concentrations of GABA in the frontal lobe have previously been associated with superior cognitive performance (111). It is theorized that reductions in frontal GABA may be contributing to the broad EF difficulties in ASD. Furthering this hypothesis is tentative support that GABA may relate to response inhibition processes (112). Evidence that the E/I imbalance can be shifted with pharmacological interventions, and that this shift is accompanied by a normalization of functional connectivity patterns in the frontal regions (113), suggests a potential intervention strategy for ASD that may lead to improvements in cognitive processes, including EF.

### Moderating Influences on EF in ASD

Moderator variables and other mediating factors (e.g. measurement of EF construct) may contribute to the observed variability of EF findings in ASD. A number of these factors are discussed below.

### Measurement of EF

The validity and reliability of EF measures may significantly moderate observed performance. Validity refers to the extent that the EF assessment tool accurately taps the theorized EF construct (36, 114). Reliability refers to the consistency of the EF assessments to measure the EF construct (36). Research in EF has been criticized for lacking valid and reliable measures. The main criticism relates to the lack of task purity in the tools utilized to measure EF (10, 64). It has been demonstrated that EF assessment tools likely measure multiple EF and non-EF processes, thus challenging their efficacy to assess distinct EFs.

Measurement of EF has traditionally focused on neuropsychological assessments sensitive to frontal lobe damage (50, 115). Assessment tools, however, including classic measures such as the Wisconsin Card Sorting Test (WCST) (115) are not pure measures of the underlying EF, e.g., set shifting (52). Experimental tasks have also been utilized as likely purer measures of discrete EFs (12). More recently, development of behavioral rating scales (37, 116) aimed to provide more ecologically valid assessments of EF (37, 117, 118) focusing on executive regulation of everyday behaviors. Studies in ASD demonstrated a significantly larger effect size for behavioral rating scales compared to neuropsychological and experimental measures (23). These findings suggest that behavioral measures may better capture EF processes and are more ecologically valid (118).

### Developmental Trajectory of EF

**Figure 1:**
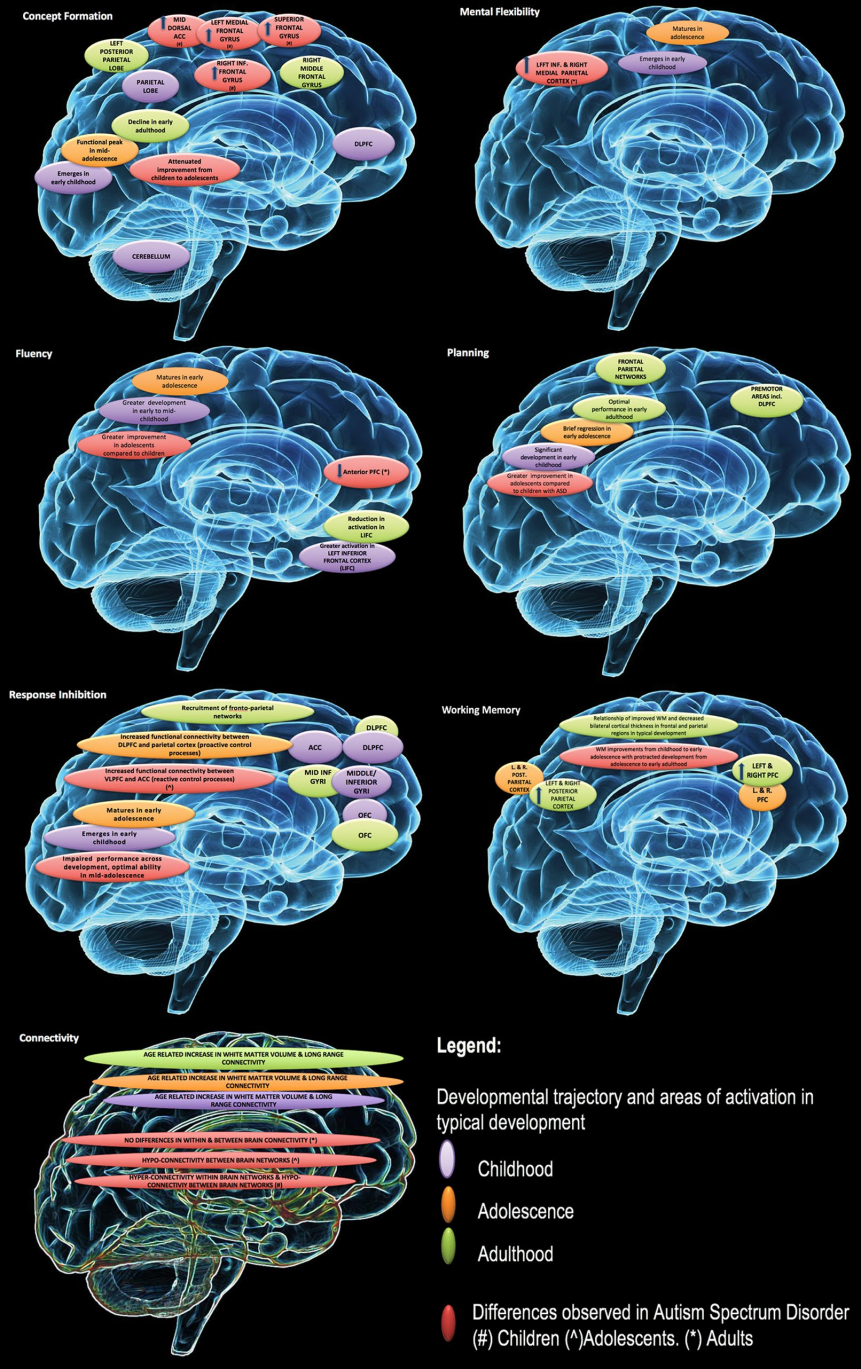
Developmental changes in executive function and associated impairment in autism spectrum disorders (ASD). Reproduced with permission from (23).

An overview of the developmental trajectory of cool EFs in neurotypical development and in ASD is presented in **Figure 1**. In typical development, maturation of EFs begins in infancy and continues throughout childhood and adolescence and into early adulthood (119, 120). The rate of improvement for individual components, with the exception of fluency, begins to taper at about age 12 (119), with most EFs reaching their peak in late adolescence/in the early 20s (120).

In ASD, there is evidence of executive dysfunction across development for discrete EF domains (e.g. working memory, set shifting/switching, fluency) (22, 68, 121) with some support of improvements in EF ability over time (122). Developmental research of hot EFs in ASD is limited. Recent research found no significant age-related changes in ASD in the neurotypical comparison group (100). This contrasts with other research in neurotypical development that suggests a variable developmental trajectory (76, 99).

The variability in peak developmental periods for distinct EFs may be contributing to some of the heterogeneity observed in EF performance in ASD. The use of mixed age groups in ASD research mask these differences and could contribute to variability observed between studies.

### Moderator Variables of EF

#### General Intellectual Functioning

Early research on EF developed partly based on observations that higher cognitive processes (e.g. planning, concept formation) may be impaired despite intact intellectual functioning. Despite this general observation, there is some empirical support that intellectual ability may moderate performance on neuropsychological assessments of EF (123). This is pertinent in the study of ASD, where differences between specific indices of intelligence (verbal, perceptual, and full-scale intelligence scales) have been reported for the clinical subgroups of autistic disorder and Asperger’s syndrome (124).

#### Sample Characteristics and Task Characteristics

Each study sets its own criteria to define eligibility and to enroll ASD participants, creating a lack of consistency between studies. These differences include ASD diagnosis (as per earlier *Diagnostic and Statistical Manual of Mental Disorders* [*DSM*] classifications), choice of comparison groups, age, and criteria matching the ASD cohort to the comparison group. The diagnostic criteria for ASD have broadened significantly since the first inclusion of autism in the *DSM-III* (125). In *DSM-5* (1), discrete diagnostic categories (autistic disorder, Asperger’s syndrome) have been merged into a single spectrum, facilitating uniformity in the diagnostic selection criteria (but likely introducing greater heterogeneity). Prior to the introduction of the *DSM-5*, a number of studies were comprised of mixed diagnostic classifications (126–129), while some studies included the informal classification of high functioning autism (HFA) (130). HFA defined ASD cohorts with no intellectual disability (IQ greater than 70). However, inclusion criteria on level of intellectual functioning ranged between studies from borderline (131), low average (129), to average (132). This could have contributed to greater variability in intellectual and executive functioning and may in part explain differences between studies.

Most studies have utilized standardized diagnostic assessments of ASD (Autism Diagnostic Observation Schedule [ADOS], Autism Diagnostic Interview [ADI]) and *DSM*-based diagnostic criteria. Some studies may utilize screening assessments (132, 133) or classification criteria not drawn from the *DSM* (134). These factors may also contribute to the variability in EF performance.

Selection of comparison control groups also varies between studies. Although most studies include neurotypical comparison groups, there have also been comparisons conducted with non-affected siblings (135, 136) or clinical groups only (137–139).

The type of assessment, whether it is a psychometric test, an experimental task, or a behavioral rating scale, is an important moderating factor in the discussion of EF. It has been suggested that behavioral rating scales capture different underlying mechanisms (140) compared to performance-based tasks and therefore should not be utilized as substitute measures of EF performance. In particular, self-ratings on behavior may reflect individual’s motivation for goal setting, achieving personal goals, and their personal expectations in relation to these goals (140). By comparison, neuropsychological assessments and experimental tasks are performance-based measures that measure EF within the designed parameters of the task. Research in ADHD (141) and ASD (20) lends some support that different cognitive mechanisms may underpin these measures. For example, low correlations were reported between performance measures and a behavioral rating scale of EF (142).

Administration format (traditional versus computerized presentations of test material) may also moderate EF performance. There is evidence that individuals with ASD perform better on computerized administration in comparison to traditional administration of EF tests (118, 143), although this is not unequivocal (144). Further, the presentation format of the test stimulus (verbal versus visual stimuli) and participant response format (motor versus verbal response) may be important moderators. This is particularly relevant to ASD research, as there is some support for superior performance in individuals with ASD in visuoperceptual tasks requiring attention to detail (145).

#### Sex Differences

ASD is a neurodevelopmental condition that occurs more in males, currently with about three males diagnosed to every one female (146). A number of theories have been proposed to explain this difference. These are based on genetic and/or neurobiological differences between males and females as described, for example, in the imprinted-X liability model (147), the male brain theory (148), and the female protective effect theory (149, 150). There is growing interest in identifying the characteristics that might differentiate male and female individuals with ASD, including EF performance. However, comparisons of males and females with ASD on neuropsychological assessments and self-/informant appraisals of EF have been limited. Some research findings (151–153) suggest differences between males and females with ASD on EF performance, while others report no differences (154, 155). One potential confounding factor is that not all studies included sex-matched neurotypical control groups. Sex differences in cognitive performance observed in neurotypical populations may also be present between females and males with ASD. These, however, will not be identified in ASD cohorts without comparisons to sex-matched neurotypical controls.

#### Co-Morbid Conditions and Affective States

The presence of co-morbid ADHD may influence EF performance in ASD, and this was particularly evident in inhibition (156). Other co-morbid conditions (e.g. depression, anxiety) have a high prevalence in individuals with ASD (157) and may have a moderating role on EF. In particular, the influence of anxiety (158) and stress (159) on EF has been well documented. Overall, research to date suggests a moderating effect of anxiety on cognitive function in non-clinical samples of highly anxious individuals (160–163). In ASD, anxiety negatively correlated with test performance on neuropsychological assessments of concept formation (18). Anxiety was also shown to correlate with impaired performance on neuropsychological measures of inhibition, mental flexibility, and shifting (164). The links between affective states and EF highlight the importance of investigating their role in ASD research.

### Heterogeneity of EF

The preceding discussion highlighted that observed executive impairment in ASD is characterized by heterogeneity with a range of contributing of factors. A research framework that can utilize EF as a marker and facilitate classification of ASD into distinct subtypes could contribute to diagnostic and intervention strategies for this group. Using cognitive and neuroimaging measures, three ASD subtypes were identified in a recent study based in part on performance on response inhibition tasks (165). A second study (166) showed that performance measures of cognitive flexibility distinguish between children with and without ASD. Interestingly, however, extension of the above study to brain connectivity circuits of cognitive flexibility did not identify subtypes at the neural level (167). The authors suggested that a dimensional approach might be more appropriate for some cognitive processes. The RDoC framework (29) incorporates a dimensional approach and can evaluate EF across cognitive and neural measures. We discuss below the efficacy of EF as an endophenotype for ASD and propose that the RDoC framework can advance research of EF in ASD.

## Executive Function as a Cognitive Intermediate Phenotype

Endophenotypes, or intermediate phenotypes (168), are characteristics that present vulnerabilities in a particular population, linking genes, brain processes, and observed behavior. Endophenotypes may encompass neurocognitive functions (136, 169), making EF a likely candidate. Criteria that must be satisfied for considering a marker as an endophenotype include: the marker must be associated with the illness/disorder in the population; it must be heritable; and it must present at higher rates within affected families than the general population (170).

The wealth of empirical findings linking EF with the broader ASD phenotype (in particular, the diagnostic clusters as defined in the *DSM-5*) support its potential as an endophenotype. Early reviews of the literature (171, 172) and empirical studies reported a correlation between neuropsychological (129, 173, 174) and behavioral measures (175) of executive impairment with severity of repetitive behaviors. This relationship was reported for specific EF domains, such as cognitive flexibility, response inhibition, and working memory. Another study suggested that EF deficits were specific to repetitive but not restricted behavior patterns (15). These findings led to theories that linked restricted and repetitive behavior symptoms to EF, suggesting that EF constructs can differentiate within behavioral clusters in ASD. A number of studies show that EF influences ToM performance in ASD (17, 176) and may influence the social communication cluster. The ToM model (5) was one of the prominent cognitive explanations for impaired social cognition in ASD. It proposed that impaired ability to attribute mental states to self and others contributes to a range of deficits including those observed in the social communication cluster (177). Recent research also indicated that ToM may predict disability (178). Support of a putative link between EF and ToM includes findings that reduced working memory moderated social communication skills (179). In summary, there is evidence that EF influences both diagnostic clusters of ASD (1) and would be a valuable endophenotype for targeted interventions.

EF has measurable behavioral outcomes (37, 142) and is linked to genetic (168) and neurobiological (180) processes. For example, functional imaging studies have demonstrated that neuropsychological assessments of EF are linked with activation of brain areas including frontoparietal (168) and frontal cortical areas (52). Further, genetic influences account for about half of the variability in EF performance (45, 168). The neural substrates of GABA and glutamate present a neural link for the EF (common factor) which has a genetic basis but may be measured with cognitive tasks (47). Lastly, there is empirical support that EF difficulties in relatives of probands with ASD are at a higher rate than the general population (181).

In summary, research on EF indicates that it satisfies the definition of endophenotypes and supports its role as an endophenotype for ASD.

## A Research Framework and Future Research Directions

We suggest that a model of EF in ASD that bridges the pathway from genetics to neural circuitry and to the observed EF phenotype may better capture the heterogeneity of EF in ASD. The unity and diversity model (12, 45, 46) provides a link for an integrated research framework for EF. The common EF factor could contribute to quantifying heterogeneity in EF performance in ASD. Complementing the above, investigation of core cool EFs (set shifting and working memory), hot EFs/behavioral regulation, and affective states in a single research framework can further advance the study of EF in ASD.

The RDoC framework (29) provides research guidelines that may resolve a number of the limitations observed in ASD research. The RDoC approach advocates a focus on a dimensional research framework. It is guided by research across “systems”-based domains that are evaluated by different levels of measurement (extending from the molecular/genetic level to the observed behavioral phenotype) (182).

The guiding principles of the RDoC framework (183) focus on: a dimensional systems approach, behavior–brain relationships, and multiple levels of analysis (molecular, circuit behavior, symptom). These principles align with the study of EF and ASD, creating a framework to guide this complex research area. Further, the RDoC framework can be adapted to reflect key characteristics of neurodevelopment (developmental trajectories/sensitive periods) (183, 184) and can be particularly relevant to the study of neurodevelopmental conditions, including ASD.

The RDoC framework presently consists of six systems domains: negative valence systems, positive valence systems, cognitive systems, social processes, arousal and regulatory systems, and sensorimotor systems. Each system is characterized by different constructs that are evaluated across distinct units of analysis (or measurement): genes, molecules, cells, circuits, physiology, behavior, self-report, and paradigms. Research of EF in ASD brings together a number of these systems and specifically the “positive valence systems,” “negative valence systems,” and “cognitive systems.”

The positive valence systems are responsible for responses to positive motivational situations or contexts, such as reward seeking. The negative valence systems are responsible for responses to aversive situations or context, such as fear, anxiety, and loss, and the cognitive systems domain is responsible for cognitive processes.

**Figure 2:**
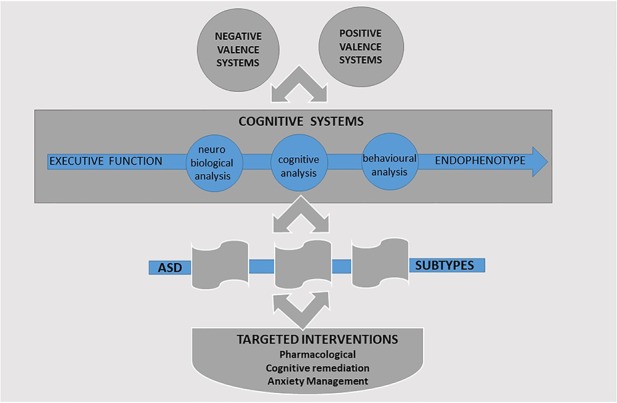
A research framework for the study of executive function (EF) in ASD.

The positive valence systems domain presents a framework for integrating the relationship between hot EFs and behavioral regulation. Complementing these, the negative valence systems domain captures the contribution of anxiety in ASD (157) and its moderating role in EF outcomes (158). Within the cognitive systems domain, the constructs of cognitive control and working memory reflect the EF factors of the unity and diversity model (12). These can evaluate the contribution of cool EFs in ASD. Investigated together, these three systems would provide researchers with a common language facilitated by a consensus on the specific components under each unit of analysis. Furthermore, such an integrated approach would provide greater opportunity to identify subtype profiles within ASD. Targeted intervention strategies can then be tailored to each profile with primary focus on the domains of the cognitive, positive, and negative valence systems. A summary of the proposed integrated framework is presented in **Figure 2**.

## Conclusion

EF is an important factor in the study of ASD and with great potential as an endophenotype. Despite the plethora of theoretical models, there is conceptual confusion in EF research that would benefit from a unified research methodology. The findings of broad EF impairment in ASD are an important step, as they unify much of the research on cool EFs and highlight that differences are likely guided by genetic variability in EF processes. The application of the RDoC framework has potential to improve our understanding of EF in ASD and elucidate the mechanisms responsible. RDoC presents a framework to integrate research obtained from diverse measures (neuropsychological tests, experimental tasks, behavioral ratings) to characterize the relevant circuitry and investigate additional factors (e.g. hot EFs) and moderators (e.g. anxiety). Taken together, the RDoC approach presents new opportunities for profiling ASD subtypes and for targeted assessments and interventions.

## Author Contributions

ED and AG contributed to the conception and planning of the review. ED conducted the literature search and provided the initial draft. ED, MD, and AG wrote sections of the manuscript. All authors contributed to manuscript revision, read and approved the submitted version.

## Funding

This work was supported by an Australian Research Council Linkage Grant (LP110200562) to AG and a NHMRC scholarship (GNT1056587) to ED.

## Conflict of Interest

The authors declare that the research was conducted in the absence of any commercial or financial relationships that could be construed as a potential conflict of interest.
